# Guided self-help Urdu version of the living life to the full intervention for secondary school adolescents with low mood and anxiety in Pakistan: A feasibility study

**DOI:** 10.1016/j.heliyon.2022.e09809

**Published:** 2022-07-08

**Authors:** Amna Khalid, Sabahat Haqqani, Christopher Williams

**Affiliations:** a Office No M27-146, Department of Family, Community Medicine and Behavioral Sciences, College of Medicine, University of Sharjah, United Arab Emirates; b Assistant Professor/ Head of the Department of Psychology, Capital University of Sciences and Technology (Previously affiliated with Fatima Jinnah Women University), Pakistan; c Honorary Senior Research Fellow (Institute of Health & Wellbeing), University of Glasgow, UK

**Keywords:** Cognitive behavioral therapy, Intervention, Adolescents, Guided self-help, Depression, Anxiety

## Abstract

There is limited evidence on the efficacy of Cognitive Behavioral Therapy (CBT) based guided self-help programs to improve low mood and anxiety in Pakistani adolescents. The aims of the current study were to assess the effectiveness of an eight week low intensity CBT-based guided self-help program, Living Life to the Full (LLTTF) on depression, anxiety and social functioning among secondary school adolescents in Pakistan. Fifty-six participants were randomly allocated to immediate (n = 28) and delayed access (n = 28) groups. Measures of depression, anxiety and social functioning were collected at baseline, post intervention and three months follow-up. There were significant improvements in measures of depression (t = -3.47, p < 0.01; *d* = 1.0), anxiety (t = -6.55, p < 0.001; d = 1.91) and social functioning (t = -4.28, p < 0.001) between immediate access and delayed access groups. These differences remained significant at three months follow-up. The study suggests that the Urdu LLTTF book course delivered in classes is effective for reducing depression and anxiety as well as improving social function among adolescents in Pakistan.

## 1 Introduction

Low mood and anxiety are common mental health problems worldwide (World Health Organization, 2017) which often start in adolescence and continue into adulthood (Copeland et al., 2009; Fergusson and Woodward, 2002; Reef et al., 2009; Ruiz and Primm, 2010; Woodward and Fergusson, 2001). Estimates of low mood and anxiety vary widely across studies and countries. In UK, 0.2–17% of adolescents' report depression (Costello et al., 2003) while 9–32% have anxiety (Essau and Gabbidon, 2013). Approximately 85% of the world's adolescents live in low- and middle-income countries (LMICs) (Department of Economic and Social Affairs, 2010) with almost no access to mental health care (Lu et al., 2018).

Pakistan is a LMIC with more than half of the population under the age of 30 and 29% between ages 15 to 29. The proportion of youth in Pakistan is higher than ever before and is expected to increase until 2050 (Ahmad, 2018). A recent community-based survey shows that 17.2% Pakistani adolescents report depression while 21.4% report anxiety (Khalid et al., 2018). There is a strong need for specialized mental health services for children and adolescents in the country (Imran et al., 2021). Community based prevention and intervention programs for depression and anxiety should be given top priory in such countries to avoid the development of the disease and its associated impairment (Wong et al., 2014).

Cognitive behavioral therapy (CBT) is recommended for the treatment of depressed (Klein et al., 2007) and anxious young people (Seligman and Ollendick, 2011). A typical CBT treatment plan involves 12–20 one hour sessions with a CBT expert. However, it remains difficult to provide specialist CBT due to the large volume of patients and severe shortage of CBT professionals. A more practical approach is highly accessible low-intensity (LI) CBT in the form of bibliotherapy (online or paper based) which can be effective in reducing symptoms of sub-threshold depression and anxiety (Musiat et al., 2014). A systematic review of meta-analyses exploring the efficacy of self-help and internet-based guided interventions indicates their effectiveness in treating depression and anxiety disorders with relatively large effect sizes (van't Hof et al., 2009). Adding further guidance and support, not necessarily by a CBT expert, in using such self-help interventions significantly improves the outcome for those with depressive and anxious symptoms (National Institute for Health and Clinical Excellence, 2004; Gellatly et al., 2007).

CBT is less available in LMICs, however limited existing data suggests that it might be effective in treating low mood and anxiety in these countries (Gellatly et al., 2007; Araya et al., 2003; Rahman et al., 2008; Patel et al., 2010) where a treatment gap of more than 90% has been reported (Wang et al., 2007). CBT in Pakistan has been effectively used for treatment of: psychosis (Husain et al., 2017); maternal depression (Rahman et al., 2008); depression and anxiety among female victims of violence (Latif and Khanam, 2017); ADHD in adolescents (Khan and Ghazib, 2017); depression and anxiety among adult patients (Irfan, 2016); depression among female university students (Zadeh and Lateef, 2012); and depression and anxiety compared with antidepressant treatment (Naeem et al., 2011).

A recent, study in Pakistan explored effectiveness of a culturally adapted Guided Self Help program based on CBT (CACBT-GSH) for treatment of social anxiety among 76 school going adolescents aged 13 to 16. Participants were randomly allocated into a control group receiving treatment as usual TAU, and an experimental group receiving TAU plus the CACBT-GSH intervention. The intervention group scored significantly lower on social anxiety and related factors encouraging the feasibility for such approaches in treating psychological illness among adolescents in Pakistan (Amin et al., 2020). The same manual has been used with patients receiving psychiatric outpatient treatment with a mean age of 31.7 years (Naeem et al., 2015). Although these studies report effectiveness of CBT for use in Pakistan in various contexts, results from the majority of these studies cannot be generalized to adolescent samples due to a limitation in terms of quality in research design and methodology (Ishfaq et al., 2014). However, all these studies emphasize using CBT based content culturally adapted for use with Pakistani population (Ishfaq et al., 2014; Naeem et al., 2010).

The present study explores the feasibility of using an adapted Urdu language version of a series of books called Living Life to the Full- LLTTF (Williams et al., 2018- current); an already available CBT-based intervention focusing on enhancing life skills. The content has been successfully used for treatment of low mood and anxiety (Williams et al., 2018; McClay et al., 2013). The 8-week, book-based CBT resources were translated and adapted for use with adolescents in Pakistan. The intervention can be delivered as written guided self-help, or via face to face or online classes. In this study they were delivered with face to face support.

The objective of the current study was to test the feasibility of using the Urdu printed guided self-help LLTTF book resources delivered via a series of live classes, with Pakistani adolescents in a school setting. The aim was to evaluate whether this is an effective treatment for low and anxious mood and to explore whether any change in rates of depression and anxiety is maintained over a three months period.

## 2 Materials and methods

The study design, ethics approval, description of the intervention, outcome measures, procedure for data collection, recruitment and allocation strategy, delivery of the intervention and statistical analyses are described in detail in this section.

### 2.1 Design

This study followed a pretest-posttest experimental design to test the feasibility of the Urdu booklet version of LLTTF for use among Pakistani secondary school adolescents.

### 2.2 Ethics

Ethical approval for the research was taken from ethics committee of Fatima Jinnah Women University. Permissions were taken from the Principals of participating schools. Participants were invited to take part in the research by sending information sheets and opt out forms to their parents. Only those given permission by their parents were invited to take part in the interventional phase of the research. The participation in the research was completely voluntary. Participants were also provided with detailed information sheets about the project. Written consent was taken from the participants.

### 2.3 Intervention

The LLTTF course used here consisted of a series of eight self-help booklets delivered via 8 classes. Each booklet focuses on a common problem faced by people experiencing depressive and anxious symptomatology affecting five areas of life: thinking, feelings, physical symptoms, behaviors and the surrounding world. The key CBT techniques incorporated in this intervention include but are not limited to thought challenging, activity scheduling and problem solving. The intervention comes with a planner and review worksheets so that participants can practice what they learn from each booklet and review their progress. The titles of the books are 1: Why do I feel so bad? 2: I can't be bothered doing anything, 3. Why does everything always go wrong? 4: I'm not good enough: (low confidence), 5: How to fix almost everything, 6: The things you do that mess you up, 7: Are you strong enough to keep your temper?, and 8: 10 things you can do to help you feel happier straight away (Williams et al., 2018).

For this study the intervention was translated into Urdu by a team of senior consultant psychologists and a linguist. The translated material was pilot tested among a small group of psychology students (11 students) to collect their feedback. Focus group discussions were carried out with these students on each translated booklet and any suggestions and feedback were discussed and incorporated. The intervention developer was closely involved throughout the process of adapting the intervention for use in Pakistan. In this context some of the examples, images, stories and analogies used in LLTTF were modified for better cultural acceptability and age appropriateness.

### 2.4 Outcome measures

A “demographic sheet” was developed for this study to record demographic details such as age, gender, education, monthly income, marital status, and family system.

A medical information sheet was designed to record the participants’ medical history such as if they had any diagnosed physical or mental illness, if they were taking any antidepressants, or receiving any interventions for the problem they report. Outcome measures were used both pre and post intervention. The Client Satisfaction Questionnaire (CSQ-8) (Nguyen et al., 1983) was administered only post intervention.

### 2.5 Patient health questionnaire – PHQ 9

The PHQ-9 (Spitzer et al., 1999) was originally developed in English and reflects diagnostic criteria for depression DSM-IV. It assesses the frequency of symptoms in the past two weeks. The item rating ranges from 0-3 for each of 9 questions (0 = not at all to 3 = almost daily) with a maximum score of 27. The total score is categorized as 0–4 = no depression, 5–9 = mild depression, 10–14 = moderate depression, 15–19 = moderately severe depression, and 20–27 = severe depression. In this study, an Urdu translated version of PHQ-9 was used which has good internal consistency, and acceptable sensitivity and specificity in Pakistani sample (Fraz et al., 2013).

### 2.6 Generalized anxiety disorder- GAD 7

The GAD-7 (Spitzer et al., 2006) is a seven item questionnaire mirroring diagnostic criteria of anxiety in DSM-IV. Each item reflects the frequency of problem in the past two weeks and responses were scored as 0 = not at all, 1 = several days, 2 = more than half the days and 3 = nearly every day. A participant can score a maximum of 21 on this scale. A Score of 0–4 indicates no anxiety, 5–9 shows mild anxiety, 10–14 reflects moderate anxiety and 15–21 is categorized as severe anxiety. An already translated and validated Urdu version was used in this study (Ahmad et al., 2017).

### 2.7 Work and social adjustment scale- WSAS

WSAS (Mundt et al., 2002) assessed the social functioning of the participants. It is made up of five items which address problems in daily routine work such as performing necessary house chores, developing and maintaining close relationships. Responses are on 0–8 likert scale with higher score indicating higher level of impairment. Scores above 20 show significant problems in social functioning. The WSAS was translated into Urdu for this research.

### 2.8 Family affluence scale FAS-II

FAS II was developed to assess the family affluence among children and adolescents (Currie et al., 1997). It has four items scored on a likert scale ranging 0–9. A score of 0–3 reflects low family wealth while a score of 4–6 shows medium and 7–9 indicates high family affluence. The scale has shown good psychometric properties as a self-report measure of material wealth among adolescents (Ravens-Sieberer et al., 2009) and has shown a strong concordance with parent report of affluence (Currie et al., 2008). The present study used an Urdu translated version of FASII (Khalid et al., 2018).

### 2.9 The client satisfaction questionnaire- CSQ-8

The CSQ-8 (Nguyen et al., 1983) was used to assess the level of satisfaction from the intervention delivered. It is an eight-item questionnaire and response ratings are on four point likert scale. It was administered at the time of post assessment as a measure of satisfaction. Scores range between 8 to 32; with higher scores indicating greater satisfaction with the intervention. It has high internal consistency *α* of CSQ-8 ranging from 0.83 - 0.93. CSQ – 8 was translated in Urdu for this research.

### 2.10 Procedure

Three nearby schools were approached for data collection. The schools were provided with a letter of ethics approval as well as support letter from the principal investigator's institution. However, they all refused to take part in the research. Reasons for refusal were hesitation from sharing student information. After the refusal, two schools were approached through personal referral by the Principle Investigators (PI) for data collection. Both schools (one girls only and one boys only) agreed to participate. However, these schools were approximately 20 km away from the PIs institute and the research assistant had to travel every day to the site of data collection. Eighth grade students were recruited for this study. This group was considered most appropriate because of their stage in education and follow-up potential.

### 2.11 Recruitment and allocation

All students in class 8^th^ in both participating schools were given opt-out forms. Students with disabilities, inability to understand the Urdu language, receiving any psychological treatment, or with active suicidal ideas were not included. For sample size estimation we followed approach used by Williams et al. (2018) for a pilot randomized controlled trial of the same intervention. We targeted a sample of 50 adolescents which is consistent with the commonly accepted sample range of 30–50 participants for pilot RCTS (Stallard, 2012). A total of 195 students in 8^th^ class who were present on the day of data collection were recruited for baseline assessment.

#### 2.11.1 Delivery

The intervention was delivered over eight weekly sessions in the form of a class led by research assistant with an MPhil in Counseling psychology who was trained in the approach and was also involved in translation of the intervention. She facilitated the study participants throughout the intervention and was supervised by a PhD Clinical Psychologist. Classes were led in a room allotted for this purpose by the school authorities. Copies of the intervention booklets were distributed each week at the start of the class. The participants would go through the booklet on their own and if they had any questions the research assistant was available throughout the sessions to respond to their queries. They were encouraged to complete the weekly worksheets to practice the skills learnt in the books. Support provided during classes was in a mixed format; (1) one to one feedback when asked, and (2) support as a group in terms of general instructions about the booklets and how to work through them. Participants were provided with the option of taking the accompanying booklets home after each session. The research assistant maintained the attendance register of the participants and made regular field notes. If a participant missed a session due to absentee, they were followed up next day.

### 2.12 Statistical analyses

Statistical Package for social Sciences Version 20 (SPSS 20) was used for data analyses. Descriptive statistics were calculated for demographic variables, depression, anxiety, and work and social adjustment for both immediate and delayed access groups. To test difference between immediate and delayed access groups, t-tests were conducted along with effect size calculations. To assess the long-term sustainability of the intervention, within group differences were estimated using a generalized linear model for Pre-intervention time, post-intervention and 3 months’ follow-up scores. Satisfaction with intervention was calculated through mean and standard deviation scores.

## 3 Results

**Figure 1:**
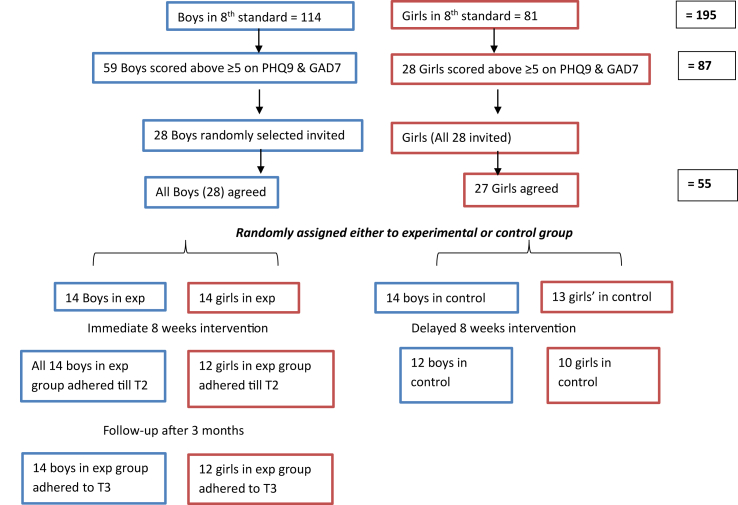
Diagram of participant flow through the intervention.

A total of 195 students who were present on the day of data collection opted into the research and were assessed with the GAD and PHQ (see Figure 1). Eighty seven (59 boys and 28 girls) students scored above the cutoff of 5 on GAD and/or PHQ. They were all invited to take part in the second phase of research. A total of 27 of 28 girls who were willing to participate in the intervention phase were randomly assigned to control (13) and experimental groups (14) using Excel software by a co-researcher who was not directly involved in intervention delivery or assessing outcomes. Of these girls 12 from the experimental and 10 from control group adhered to the intervention. 28 of 59 boys who were willing to participate in the intervention phase were then randomly assigned to control (14) and experimental groups (14). Therefore, 26 of the 28 participants assigned into immediate and 22 of 27 assigned to delayed access group adhered to the intervention. Cronbach's alpha for PHQ-9 in the present study was 0.75. Cronbach's alpha for GAD-7 in the present study was 0.83. Cronbach's alpha for WSAS in the present study was 0.77.

The sample has a good representation of both boys and girls. Almost all the participants had working fathers while mothers were homemakers. About 70% of the data identified with low socio-economic strata (see Table 1).

**Table 1:** Demographic characteristics of categorical variables across immediate access and control group (n = 55).

Characteristic		Immediate access group (n = 26) f(%)	Delayed access group (n = 22) f(%)
Gender∗			
	Female	12(46.2)	10(45.5)
	Male	14(53.8)	12(54.5)
Parental Occupation			
Father∗	Working	22(95.7)	17(73.9)
	Non-working	1(4.3)	3(13)
Mother∗	Working	1(3.8)	2(9.1)
	Housewife	24(92.3)	17(73.9)
Relationship status∗			
	Single	26(100)	18(90)
	Engaged	0	2(10)
Family affluence∗			
	Low (0–3)	18(69.2)	14(69.9)
	Middle (4–6)	8(30.8)	7(30.4)
	High (7–9)	0(0)	1(4.3)

∗ missing data.

### 3.1 Effectiveness of the intervention

**Figure 2:**
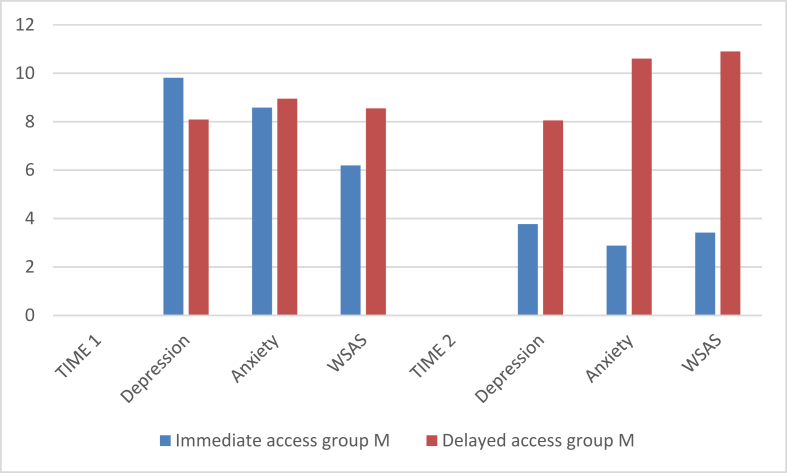
Mean scores on the different measures for both groups before and after the intervention at T1 and T2.

Table 2 and Figure 2 show that at pre-intervention both experimental and control groups did not differ in depression and anxiety symptoms. There was a significant reduction in symptoms of depression and anxiety in experimental group after receiving the intervention (Time 2).

**Table 2:** Comparison of depression and anxiety scores of Immediate access group and Delayed access group (n = 48).

	Immediate access group		Delayed access group		t(p)	*d*
	M	SD	M	SD		
TIME 1						
Depression	9.81	4.55	8.09	4.25	1.34(>0.05)	
Anxiety	8.58	5.32	8.95	5.61	-0.24(>0.05)	
WSAS	6.19	6.41	8.55	7.64	-1.44(>0.05)	
TIME 2						
Depression	3.77	4	8.05	4.52	-3.47(<0.01)	1.0
Anxiety	2.88	3.02	10.6	4.83	-6.55(<0.01)	1.91
WSAS	3.42	4.62	10.9	7.01	-4.28(<0.01)	1.26

### 3.2 Effectiveness of intervention over 3 month's periods

Table 3 summarizes the results using a Generalized Linear Model which was constructed to test the variation in scores of respondents with respect to time (pre, post and 3 months). It was hypothesized that the depression, anxiety, and work and social adjustment scores of respondents will decrease significantly from pre-intervention time and will remain significantly lower after 3 months' time. Scores of each respondent were compared across time 1, 2 and 3 (pre, immediate post (8 weeks) and 3 months respectively). The results indicate that there is significant improvement in depression, anxiety, work and social adjustment scores from time 1 to time 2, and this difference in symptoms remains significant from pre intervention till 3 month follow-up, showing the effectiveness of intervention in reducing symptoms after intervention and sustaining the reduction in symptoms after 3 months’ time.

**Table 3:** Within group differences estimated from generalized linear models from Pre-intervention time to post-intervention and 3 months’ follow-up.

Response	Time	Difference from baseline			
		Estimate	95% CI		*p*
			LL	UL	
Depression	Post intervention	-0.96	-1.19	-0.72	<0.01
	3 months follow-up	-1.08	-1.32	-0.83	<0.01
Anxiety	Post intervention	-1.09	-1.35	-0.83	<0.01
	3 months follow-up	-1.09	-1.33	-0.83	<0.01
Work & social adjustment	Post intervention	-0.59	-0.85	-0.33	<0.01
	3 months follow-up	-0.54	-0.79	-0.28	<0.01

### 3.3 Satisfaction from the intervention

Participants showed a high level of satisfaction from the intervention and its delivery at post-intervention and at three months’ follow-up (Table 4). Out of a total of 32, on average 27.1 mean score on CSQ-8 was reported by the IAG as well as the DAG at post-intervention and 3 months follow up by IAG.

**Table 4:** Participants’ satisfaction from the intervention at various stages.

	M	SD
Immediate access group satisfaction post-intervention (n = 26)	27.08	4.39
Immediate access group satisfaction 3 months follow-up (n = 26)	27.12	4.55
Delayed access group satisfaction from the intervention (n = 23)	27.30	5.44

## 4 Discussion

The aim of the present study was to evaluate the feasibility of using the Urdu version of the LLTTF intervention (books taught in a series of classes) for adolescents with depression and anxiety symptoms. On the primary outcome measures, we found effect sizes of 1 or greater in favor of the immediate treatment access group at post-treatment. The immediate treatment access group maintained lower scores on all measures at 3 months’ follow-up compared to pre-intervention. Participants reported high satisfaction from the intervention.

In terms of the feasibility of delivering LLTTF in class format in school setting multiple observations were made. Initially three nearby schools were approached for participation on a convenience basis. All three refused to participate giving no clear reasons. When approached via a personal reference of the PIs two schools agreed. However, once the schools with pre-established contacts agreed to participate they extended their support throughout the project by arranging appropriate venue for conducting classes, sticking to the study protocols and taking care of the needs of the research assistant. The reasons for initial refusal could be because it is not usual in Pakistan to approach community samples like schools for research purposes. This is corroborated by a very low rate of research publications from the country (Irfan, 2010). Cultural perceptions of illness and healthcare or lack of understanding may be another plausible explanation which has been seen as a barrier to data collection in LMICs (Ritchie et al., 2016). Furthermore, the sociopolitical scenario of Pakistan, make the head of an institute hesitant to allow a stranger regular entry to their building. Schools with pre-established contacts agreed to participate because, being a collectivistic society Pakistanis show reverence for friends, family and acquaintances and prior contact or reference may provide a sense of trust and security. Another reason could be that the schools refusing to participate were initially accessed for permission by the research assistant rather than the PIs while those who agreed to participate were approached directly by the PIs through an established contact. This could result into principals of the refusing schools taking the purpose casually. Similar findings were reported by another study conducted in LMICs where intervention uptake was poor because patients lacked trust in lower-cadre health care workers (Ritchie et al., 2016).

Difficulties were also faced with sending female research assistant to the boy's school. Therapist's gender has been considered a significant aspect of therapy. Both male and female clients report improved therapeutic alliance with female therapists (Bhati, 2014; Lambert, 2016). The role of the research assistant in the present study was only to provide support and guidance. In future, this could be explored by hiring male research assistants for collecting data from male participants to test if this increases engagement and access to schools which prohibit intersex interactions. This information will provide baseline for potential future large-scale clinical trials in context of Pakistan or similar cultural settings. Another issue was travelling long distances every day to collect data which was difficult for a female research assistant. Challenges like difficulty gaining access to the study site and travelling long distances for data collection have been reported earlier in LMICs (Nel et al., 2017). However, data in this study was collected primarily from underage students after thorough review and adaptation of the intervention for age –appropriateness and cultural sensitivity, therefore, this challenge was manageable. Future research should keep this aspect of feasibility in mind before planning data collection. We recommend that future researchers develop field contacts before they start data collection as it may delay the data collection process. Furthermore, hiring gender matched data collectors may ease the collection process.

Overall, consent and recruitment rates were high (98%) demonstrating an appetite from both pupils and their parents to participate in this study. A high response rate of completed questionnaires (100% of consenting participants) was obtained at baseline. This reduced at follow up yet remained at a reasonable level (89.1%), by which time six pupils had dropped out from the study due to changed circumstances. The dropout rates at 3 months’ follow-up was 0% for the immediate access group. CBT usually has a good response rate as compared to other treatments as reported in a recent meta-analysis (Hofmann et al., 2012). However, in a recent study exploring guided self-help CBT based intervention among adolescents for treatment of social anxiety in Multan city of Pakistan, the refusal rate was 27.4% (Amin et al., 2020). High adherence rate in the current study could be because participants were never exposed to such intervention and it was their unique chance to seek help. This aspect needs to be explored further. It should be noted that a teacher from the school introduced the study and there may have been feelings of obligation to participate. However, the voluntary nature of participation was explicitly emphasized to participants and their parents via an information sheet by the research team.

In terms of the effectiveness of the intervention, between group and within group differences are encouraging with more than 5 points (demonstrating a clinically significant change in severity category) decrease in scores on measures of depression and anxiety post intervention. This reduction in scores is corroborated in a previous study among community-based adults in the UK (Williams et al., 2018).

The efficacy of the intervention was ascertained by comparing participants' scores from T1 to T2 and T3. The intervention successfully reduced participants' scores on measures of depression and anxiety and this effect remained significant at three months’ follow-up. The results on the effectiveness of the intervention are in line with a previous study pilot testing the English version of the same intervention in Scottish secondary school setting (Mackenzie, 2016). Although CBT has demonstrated favorable long-term effectiveness in youth in community sample (Kodal et al., 2018), the effectiveness of LLTTF over the long term needs to be ascertained.

The intervention was also successful in improving participants' ability to perform their daily work and enhanced their social adjustment over time. This is one of the core components of CBT that it focuses not only on people's view of themselves but also of others and the world around. Such treatment effects on social adjustment have been reported previously (Suveg et al., 2017).

## 5 Strengths and limitations

This study aimed to test the feasibility of delivering the LLTTF resources (books + class support) using a robust methodological design, but it carries some limitations. Our overall experience of intervention delivery in a school setting is positive and suggests it is possible for trained individuals to provide support for guided self-help mental health interventions within the school, and the majority of pupils and parents accept this provision. However, it should be noted that this school is located in the suburban area of Islamabad, capital city of Pakistan. Therefore, it is difficult to ascertain whether the feasibility would be as good or would generalize to rural, ethnically diverse schools in more deprived areas.

Strengths were the random allocation to control and intervention groups. However, the sample size of the study was low. As intervention and control groups were from same school this could result in contamination of information across groups. Problems were encountered initially to get access from schools. This study used the adult version of the resource intervention. Although no major differences are present in adolescents and adult version, using the age appropriate version might be more feasible. The study used paper print version of the intervention, future research may use the booklets with modified illustrations from the UK version which may be more interesting for younger participants. It is further recommended that future studies use a mixed methods design and longer follow-up to ascertain the effectiveness of the intervention.

## 6 Conclusion

To conclude, the pilot data shows that the guided self-help Urdu version of LLTTF was effective in reducing symptoms of low mood and anxiety in Pakistani adolescent's sample. For this age group, class/small group format was easy to manage within the school environment. The intervention was easy to be delivered through a trained graduate in psychology.

## Declarations

### Author contribution statement

Dr. Amna Khalid: Conceived and designed the experiments; Performed the experiments; Analyzed and interpreted the data; Contributed reagents, materials, analysis tools or data; Wrote the paper.

Dr Sabahat Haqqani: Analyzed and interpreted the data; Contributed reagents, materials, analysis tools or data; Wrote the paper.

Dr Christopher William: Conceived and designed the experiment; Wrote the paper.

### Funding statement

This work was supported by Higher Education Commision, Pakistan [21-910/SRGP/R&D/HEC/2016].

### Data availability statement

The data that has been used is confidential.

### Declaration of interests statement

The authors declare no conflict of interest.

### Additional information

No additional information is available for this paper.

## Appendix A Supplementary data

questionnaire questionnaire
