# Corrigendum to “Guided self-help Urdu version of the living life to the full intervention for secondary school adolescents with low mood and anxiety in Pakistan: A feasibility study” [Heliyon 8 (7) (2022) e9809]

**DOI:** 10.1016/j.heliyon.2022.e10593

**Published:** 2022-09-13

**Authors:** Amna Khalid, Sabahat Haqqani, Christopher Williams

**Affiliations:** a Office no M27-146, Department of Family, Community Medicine and Behavioral Sciences, College of Medicine, University of Sharjah, United Arab Emirates; b Head of the Department of Psychology, Capital University of Sciences and Technology, Pakistan; c Institute of Health & Wellbeing, University of Glasgow, UK

In the original published version of this article, the authors declared no conflicts of interest. They later clarified that one of the authors is a shareholder in the company that provided material resources for the conduction and publication of the study. The Editor notes that the article has been additionally assessed given the new conflict of interest and its correctness has been confirmed. The authors apologize for the errors. Both the HTML and PDF versions of the article have been updated to correct the errors.

## Declaration of interest statement

Professor Williams is principle author of the educational life skills books and worksheets used in this project. He is Director of Five Areas, a Limited Company that commercialises these resources, and of which Professor Williams is a shareholder. Five Areas Ltd part-contributed to the journal publication costs. No other authors declare conflicts of interest.

