# Supplementary material for: Guided self-help Urdu version of the living life to the full intervention for secondary school adolescents with low mood and anxiety in Pakistan: A feasibility study
Source: Heliyon. 2022 Jul 8;8(7):e09809. doi: 10.1016/j.heliyon.2022.e09809 (PMC9293591; doi:10.1016/j.heliyon.2022.e09809)
Supplement: questionnaire [file mmc1.docx]

GAD-7

Over the last two weeks, how often have you been bothered by the following problems?

Not at all 0

Several days 1

More than half the days 2

Nearly every day 3

1. Feeling nervous, anxious, or on edge

0 1 2 3

2. Not being able to stop or control worrying

0 1 2 3

3. Worrying too much about different things

0 1 2 3

4. Trouble relaxing

0 1 2 3

5. Being so restless that it is hard to sit still

0 1 2 3

6. Becoming easily annoyed or irritable

0 1 2 3

7. Feeling afraid, as if something awful might happen

0 1 2 3

PHQ-9

Over the last 2 weeks, how often have you been bothered by any of the following problems?

Not at all 0

Several days 1

More than half the days 2

Nearly every day 3

Little interest or pleasure in doing things

0 1 2 3

2. Feeling down, depressed, or hopeless

0 1 2 3

3. Trouble falling or staying asleep, or sleeping too much

0 1 2 3

4. Feeling tired or having little energy

0 1 2 3

5. Poor appetite or overeating

0 1 2 3

Feeling bad about yourself or that you are a failure or have let yourself or your family down

0 1 2 3

Trouble concentrating on things, such as reading the newspaper or watching television

0 1 2 3

Moving or speaking so slowly that other people could have noticed. Or the opposite being so figety or restless that you have been moving around a lot more than usual

0 1 2 3

Thoughts that you would be better off dead, or of hurting yourself

0 1 2 3

**Work and Social Adjustment Scale- WSAS**


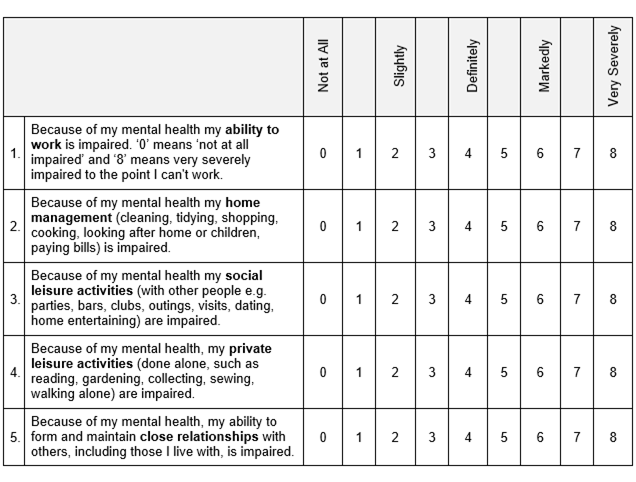


Client satisfaction questionnaire

1. How would you rate the quality of the services you have received?

2. Did you get the kind of service you wanted?

3. To what extent has our program met your needs?

4. If a friend were in need of similar help‚ would you recommend our program to him or her?

5. How satisfied are you with the amount of help you have received?

6. Have the services you received helped you to deal more effectively with problems/difficulties?

7. In an overall general sense‚ how satisfied are you with the services you have received?

8. If you were to seek help again‚ would you come back to our program?

1=Poor‚ 2=Fair‚ 3=Good‚ 4=Excellent
